# What role does mindfulness play in regulating fear of COVID-19 and associated mental health? The results of a cross-sectional study

**DOI:** 10.3389/fpsyg.2022.969087

**Published:** 2023-01-19

**Authors:** Kunhua Lee, Yu-Xuan Lee, Yu-Tung Cheng

**Affiliations:** Department of Educational Psychology and Counseling, National Tsing Hua University, Hsinchu, Taiwan

**Keywords:** pandemic, fear of COVID-19, five facet mindfulness questionnaire, orientation to experience, negative emotions

## Abstract

**Objectives:**

The pandemic has greatly impacted people’s lives and mental health. Therefore, it is now especially important to help people maintain good mental health. The positive effects of mindfulness-based practices on mental health have been demonstrated previously. However, no consensus has yet been reached on the potential mechanisms of mindfulness. This study adopted the two-component model of mindfulness to explain the relationships between fear of COVID-19, and mental health. We proposed the following hypothetical model: (1) fear of COVID-19 could affect orientation to experience; (2) orientation to experience could affect mental health. Directly; (3) fear of COVID-19 could mental health directly; (4) orientation to experience could be a mediator between fear of COVID-19 and mental health.

**Methods:**

We conducted an online survey in the present study. Three hundred and forty-four respondents were recruited to participate in the present study. After informed consent, they completed the questionnaires on the websites. The participants were asked to complete a questionnaire including the Beck Depression Inventory-II, Beck Anxiety Inventory, the Taiwan version of the Five Facet Mindfulness Questionnaire, and the Fear of COVID-19 Scale. Descriptive analysis and structural equation modeling were used to analyze the data and examine the goodness-of-fit indices.

**Results:**

Our results not only showed orientation to experience playing as a mediator between fear of COVID-19 and mental health; but also confirmed the roles of nonjudgment and nonreactivity in regulating emotions.

**Conclusion:**

Experimentation and longitudinal study could be applied to examine the roles of nonjudgment and nonreactivity in the future.

## 1. Introduction

The COVID-19 pandemic has had significant impacts worldwide. World Health Organization (WHO) COVID-19 dashboard, there had been 625,248,843 cases of COVID-19 globally by the time of this submission. The pandemic outbreak caused a substantial economic shock and strongly affected people’s employment, health, relationships, and mental state (Goodell, 2020). The rapid spread of this constantly mutating virus made people feel uncertain about their health (Watkins, 2020), and such a sense of uncertainty can result in long-term distress. Facing unemployment and random lockdowns, increasing numbers of people reported feeling moderate to severe depression or anxiety due to the pandemic (Cullen et al., 2020; Pfefferbaum and North, 2020).

Meanwhile, COVID-19 pandemic makes people isolated from others to avoid infected diseases, and then people feel more anxiety, loneliness, and depression (Robb et al., 2020). Besides, lockdowns due to the pandemic could increase the prevalence of conflict in families (Piquero et al., 2021). In line with previous studies, people need to spend more time learning alternative skills for these obstacles of the pandemic. More people could not adjust well and decided to commit suicide during the pandemic (McIntyre and Lee, 2020). Besides adults, the literature also emphasized the negative impacts of the pandemic on children; more children reported anxiety, depression, and post-traumatic symptoms during the pandemic (de Miranda et al., 2020). Due to the pandemic, mental health issues are emerging and need to be understood and discussed.

The outbreak of the pandemic has impacted both psychological and physical health. In addition to being provided with accurate information about the pandemic and the virus, people should be taught more effective approaches to self-care to ease their stress (Daniel, 2020). Despite understanding the effectiveness of face masks and personal hygiene in preventing infection with COVID-19 (Omer et al., 2020), people still fear the presence of COVID-19 in their daily lives (Ahorsu et al., 2022). Anxiety and depression could have shared risk factors, such as rumination, attentional bias, or emotional regulation (Sloan et al., 2017). Therefore, the symptoms of anxiety and depressive symptoms could be improved by evidenced emotional regulations. Literature indicated that mindfulness-based practice could effectively improve anxiety and depressive symptom in literature (Arch and Craske, 2006; Hofmann et al., 2010).

### 1.1. Mindfulness

*Mindfulness* is defined as purposefully paying attention to one’s experience, moment by moment, nonjudgmentally (Ludwig and Kabat-Zinn, 2008). Some kinds of mindfulness-based practice could need to intentionally engage with sensations and shift one’s attention from the outside to the inside, such as breath meditation or body scan. As thoughts and feelings emerge, they are treated nonjudgmentally and objectively (Shapiro et al., 2006). Literature indicated mindfulness is not only a “state”; it could also be treated as a trait (Shapiro et al., 2011). Through mindfulness-based practice, mindfulness, an inherent human capacity, could be enhanced and cultivated (Shapiro et al., 2011; Kiken et al., 2015).

The construct of mindfulness can be classified into five facets: nonreactivity to inner experience, observing, acting with awareness, describing, and nonjudging of inner experience (Hill and Updegraff, 2012). Literature indicated that observing had a lower loading of mindfulness, and a possible explanation is the effect of meditation experience (Aguado et al., 2015). However, the literature indicated five-factor model could not be stable based on several pieces of evidence; for example, a cross-sectional study indicated that describing, awareness, nonjudgmentally, and non-reacting were negatively associated with anxiety and depression except for observing (Deng et al., 2011). Another study also confirmed the effects of nonjudgmentally and awareness on depression; only nonjudgmentally also could affect anxiety (Cash and Whittingham, 2010). In sum, the relationship between the five-factor model of mindfulness and mental health should be reconsidered or re-examined in the future.

The two components model of mindfulness was applied in the present study for two reasons; one is the two-component model of mindfulness developed from the definition of mindfulness by Kabat-Zinn (Bishop et al., 2004); another reason is that the concept of mindfulness could be covered by the two-component model of mindfulness (Bishop et al., 2004). Another reason is that the concept of mindfulness could be covered by the two-component model of mindfulness (Brown and Ryan, 2004).

Two-component model of mindfulness and mental health. Based on the two-component model, self-regulation of attention is focused on observation and awareness. People must change focus and shift their attention to their bodies and mind. The second component is the orientation to experience, where people learn to accept their feelings and thoughts in a nonjudgmental way and where practicing mindfulness with curiosity can cultivate other mindfulness; besides, compared to self-regulation of attention, emotional regulation can occur through the cultivation of awareness of inner experience, which is termed the stage of orientation to experience (Bishop et al., 2004).

The two-component model of mindfulness also was applied to explain the relationship between mindfulness and mental health, for example, the researchers found self-regulatory attention had a direct effect on depression, and orientation to experience had a direct effect on depression and anxiety (Tran et al., 2014). However, the participants in the previous study were experienced meditators. Naïve community populations were not recruited in the previous study. Moreover, the purpose of the present study examined the relationship between two components of mindfulness and mental health in a population of naïve community populations.

Furthermore, depression and anxiety could be improved when people shift their attention from outside to inside and experience their feeling and sensation openly. When people with depressive moods stay models, they are more easily able to relieve their suffering (Kostanski and Hassed, 2008). Greater contact and actual use of mindfulness-based practice could predict increased mindfulness. People with depressive symptoms could accept and experience depressive symptoms without judgment (Breedvelt et al., 2019; Strohmaier, 2020). Another study used the Five Facet Mindfulness Questionnaire (FFMQ) to examine the effect of the two-component model on anxiety and depression. It showed that depression was strongly related to acting with awareness, nonjudging of inner experience, and nonreactivity to inner experience, but not significantly to the observing or describing facets (Bohlmeijer et al., 2011).

Observing was only strongly related to psychological adjustment and well-being of inexperienced mediators, possibly because of changes in how the attention is focused rather than changes in emotion (Baer et al., 2008; Huang et al., 2015). Additionally, nonjudging of inner experience and nonreactivity to inner experience was also referred to as switching from the “doing” model to the “being” model, which could reinforce the component of orientation to experience (Bishop et al., 2004; Chambers et al., 2009).

**Figure 1:**
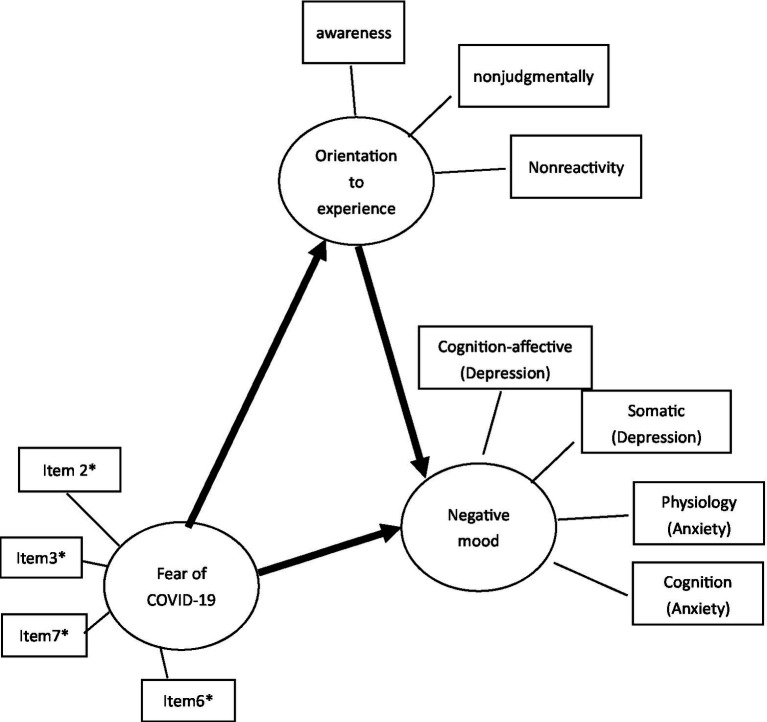
Hypothesized model. *Item 2: It makes me uncomfortable to think about coronavirus-19. *Item3: My hands become clammy when I think about coronavirus-19. *Item6: I cannot sleep because I’m worrying about getting coronavirus-19. *Item7: My heart races or palpitates when I think about getting coronavirus-19.

During the pandemic, most people are afraid of contracting COVID-19 because of its moderate to severe symptoms (such as fever, dry cough, and dyspnea). Therefore, fear of exposure to COVID-19 and related consequences have impacted people’s daily lives and emotional statuses (Fitzpatrick et al., 2020). Due to several reasons, including the literature supported the effect of orientation to experience on mental health, the facets of orientation to experience were highly related to mental health, and observing self-regulatory attention on mindfulness was still inconsistent, the present study would focus on the effect of orientation to experience, and proposed a hypothesized model of mindfulness (orientation to experience) regarding fear of COVID-19 (see Figure 1).

Several hypotheses are proposed: (1) fear of COVID-19 would have a direct effect on mental health; (2) fear of COVID-19 would have a direct effect on the facets of orientation to experience; (3) the facets of orientation to experience (acting with awareness, nonjudging of inner experience, and nonreactivity to inner experience) would have a direct effect on mental health; (4) the facets of orientation to experience could mediate between fear of COVID-19 and mental health.

## 2. Materials and methods

### 2.1. Procedures and participants

This study was conducted from January 2020 to December 2020, we used a cross-sectional study design, and was approved by the institute review board of National Tsing Hua University (REC no. 11003HT032). Participants were recruited *via* our website and social networking sites. After providing informed consent on the website, the participants completed an online questionnaire. Each participant received NT$ 50 after completing the questionnaire. Their responses were saved online until the end of the study.

The criteria for recruitment were an age of at least 20 years, the ability to read and write, the ability to use the internet, and an educational level of at least elementary school completion. Exclusion criteria were poor reality-test results, vivid psychotic symptoms, delirium, and dementia. A total of 334 participants were recruited and completed the questionnaire.

### 2.2. Measurements

Personal information: We collected the following personal data: age, gender, education level, occupation, marital status, and perceived health.

Fear of COVID-19: We used the scale that was developed for assessing fear of COVID-19. The scale included seven items, which were rated using a five-point Likert scale (1 denoted “strongly disagree,” 2 denoted “disagree,” 3 denoted “neither agree nor disagree,” 4 denoted “agree,” and 5 denoted “strongly disagree”). Higher scores indicated a greater fear of COVID-19 (Ahorsu et al., 2022). The example items of COVID-19 included It makes me uncomfortable to think about coronavirus-19 (item2) or My hands become clammy when I think about coronavirus-19 (item3). However, the internal consistency and reliability (Cronbach’s α) were not sufficient in this study (α = 0.31), but after deleting the items with lower variance (items 1, 4, and 5), Cronbach’s α was 0.70.

Mindfulness: The Taiwan version of the FFMQ (T-FFMQ) was used to assess the extent of mindfulness; in it, 39 items were rated using a five-point Likert scale (1 denoted “never,” 2 denoted “occasionally,” 3 denoted “sometimes,” 4 denoted “often,” and 5 denoted “always”; Huang et al., 2015). Example items of mindfulness included “I am easily distracted by other things” or “I have difficult describing my feeling.” To test our hypothesized model, we included orientation to experience in the model. The latent variables of orientation to experience from the T-FFMQ were the facets of nonjudging of inner experience, acting with awareness, and nonreactivity to inner experience (Christopher et al., 2012). In this study, Cronbach’s α for acting with awareness was 0.64. For nonjudging of inner experience, Cronbach’s α was only 0.35, so we removed three items of the nonjudging facet from the reliability test (items 3, 14, and 39). Cronbach’s α for the revised nonjudging facet was 0.67. For nonreactivity to inner experience, Cronbach’s α was 0.24, so we removed three items of the nonreactivity facet from the reliability test (items 4, 24, and 33). Cronbach’s α for the revised nonreactivity facet was 0.54.

Mental health: We used the Beck Depression Inventory-II (BDI-II) and the Beck Anxiety Inventory (BAI) to assess the severity of depression and anxiety, respectively. The BDI-II comprised 21 items that were rated using a four-point scale (Dozois et al., 1998). Higher scores indicated more-severe symptoms of depression. The BDI-II had two subscales: cognitive–affective and somatic (Dozois et al., 1998). Their reliability was 0.88 and 0.58, respectively. The BAI comprised 21 items that were also rated using a four-point scale, with a total possible score range of 0–63. Higher scores indicated more-severe symptoms of anxiety (Beck et al., 1993). The BAI had two subscales: physiological and cognitive. Their reliability was 0.83 and 0.86, respectively.

### 2.3. Statistics

Descriptive analysis is used to present the distributions of the demographic data and measured variables. We used structural equation modeling (SEM) to examine the goodness-of-fit of the hypothesized model by AMOS 18.0. The maximum likelihood method was to estimate the goodness of fit. Meditation analysis was examined by the Sobel test. The goodness-of-fit indices we used were the comparative fit index (CFI; > 0.95); root means the square error of approximation (RMSEA; < 0.06); and standardized root mean square error (SRMSR; < 0.08; Nye and Drasgow, 2011). Statistical significance was assumed at *p* < 0.05.

## 3. Results

### 3.1. Distribution of demographic data and measured variables

Table 1 shows the distribution of demographic data. 68% of the participants were 20–29 years old; 69.5% of the participants were females; Almost 90% of the participants were graduated from college or higher education; 60.8% of the participants were homemakers; More than 90% of the participants reported to feel healthy and well subjectively. Table 2 shows the correlations among the measured variables. All the measured variables were significantly correlated with each other, except for acting with awareness. Acting with awareness was only significantly correlated with nonjudging of inner experience and fear of COVID-19.

**Table 1:** Distributions of demographic and measured variables.

Variables	N/%
Gender	
Male	102/30.5
Female	232/69.5
Age	
20–29 years	227/68
30–39 years	28/8.4
40–49 years	25/7.5
50–59 years	52/15.6
≥ 60 years	2/0.6
Educational level	
Elementary	1/0.3
Junior high school	1/0.3
Senior high school	32/9.6
Undergraduate	247/74
Graduate	53/15.9
Occupation	
Police or soldier	0/0
Government employee	9/2.7
Education	13/3.9
Business	18/5.4
Labor	20/6
Farming	25/7.5
Medicine	23/6.9
Service industry	10/3.0
Homemaker	203/60.8
Student	5/1.5
Retired	3/0.9
Other	5/1.5
Marital status	
Married	85/25.4
Single	243/72.8
Divorced	4/1.2
Separated	0/0
Widowed	2/0.6
Subjective health status	
Feel healthy and well	306/91.6
Feel unhealthy	28/8.4
Feel unhealthy and need help	0/0
Feel unhealthy and need more help	0/0

**Table 2:** Correlations among measured variables.

	1	2	3	4	5	6	7	8
1	–							
2	−0.38**	–						
3	−0.01	−0.41**	–					
4	0.03	0.19**	−0.21**	–				
5	0.04	0.35**	−0.28**	0.68**	–			
6	−0.01	0.38**	−0.32**	0.48**	0.61**	–		
7	−0.07	0.30**	−0.17**	0.42**	0.45**	0.59**	–	
8	0.03	−0.60**	0.54**	−0.22**	−0.32**	−0.46**	−0.29**	–
Mean	3.097	2.522	3.386	0.165	0.36	0.40	0.728	3.43
SD	0.576	0.626	0.650	0.263	0.405	0.371	0.592	0.72

1: acting with awareness; 2: nonjudging of inner experience; 3: nonreactivity to inner experience; 4: physiological subscale of BAI; 5: cognition subscale of BAI; 6: cognition–affective subscale of BDI-II; 7: somatic subscale of BDI-II. 8: fear of COVID-19; *p < 0.05; **p < 0.01; ***p < 0.001.

### 3.2. The goodness-of-fit indices for the hypothesized model

**Figure 2:**
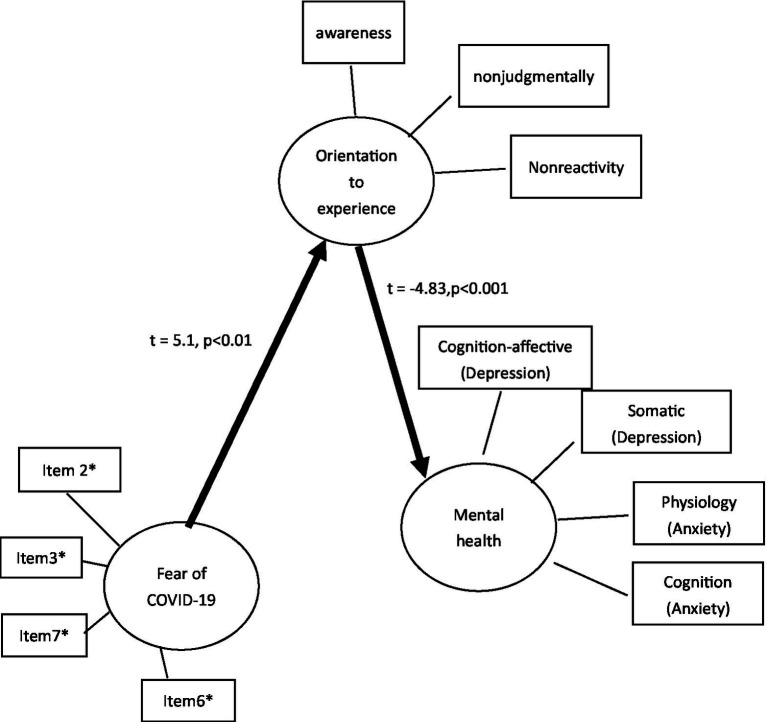
The coefficients of the revised hypothesized model. *Item 2: It makes me uncomfortable to think about coronavirus-19. *Item3: My hands become clammy when I think about coronavirus-19. *Item6: I cannot sleep because I’m worrying about getting coronavirus-19. *Item7: My heart races or palpitates when I think about getting coronavirus-19.

Figure 2 shows the goodness-of-fit indices for the hypothesized model, which were χ2 = 215.76, *p* = 0.000, CFI = 0.87, and RMSEA = 0.113. Two paths of the hypothesized model were not statistically significant, including the coefficient of the path from fear of COVID-19 to mental health (−−0.14; *p* = 0.89). The coefficient of the path of fear of COVID-19 to orientation to experience was 5.59 (*p* < 0.001). The results of the Sobel test for mediation analysis showed nonjudgmentally (*t* = −6.70. *p* = 0.00) and nonreactivity (*t* = −4.986, *p* < 0.001) could be the mediators between the fear of COVID-19 and mental health, whereas awareness could not be.

### 3.3. Revised hypothesized model

Based on the goodness-of-fit indices for the hypothesized model, we removed the nonsignificant path of fear of COVID-19 to mental health. In the revised model, all paths were statistically significant, including the path of fear of COVID-19 to orientation to experience (*t* = 5.1, *p* < 0.001) and the path of orientation to experience to mental health (*t* = −4.83, *p* < 0.001). The goodness-of-fit indices for the revised model were χ^2^ = 115.67, *p* = 0.000, CFI = 0.904, RMSEA = 0.104, and SRMR = 0.06 (see Figure 2).

## 4. Discussion

In this study, we proposed a hypothesized model examining the effect of mindfulness on mental health. We found that orientation to experience could function as a mediator between fear of COVID-19 and mental health.

Our results supported the hypothesis that fear of COVID-19 is strongly related to mental health. COVID-19 is highly infectious and was initially associated with high mortality rates. Therefore, people were extremely worried about being exposed to COVID-19 in public situations. Other studies also support the relationship between fear of COVID-19 and poor mental health outcomes (anxiety and symptoms of depression; Şimşir et al., 2022). Therefore, it is very important to develop effective treatments to ameliorate the fear of COVID-19.

Our results also found a negative relationship between COVID-19 scales, and BDI or BAI. We think of several possible explanations. One is that the construct of COVID-19 could not be similar to BDI or BAI, and the nature of fear of COVID-19 could be like the extent of perception and the possible consequences of COVID-19. Therefore, people could believe the items in the fear of COVID-19 scale could include the extent of personal percept and appreciation of the situations of COVID-19. Moreover, people could not feel anxiety or depression due to the pandemic when they begin to appreciate objectively (Bressington et al., 2020). Further studies could experiment to examine the content and nature of the fear of COVID-19. Additionally, the fear of COVID-19 was positively related to awareness, there was no significant level of correlation. Nonreactivity was positively associated with the fear of COVID-19. In other words, they began to experience the fear of COVID-19. Maybe a longitudinal study or experimentation could be conducted in the future.

We hypothesized that fear of COVID-19 would be negatively correlated with the facets of orientation to experience. Despite the existence of significant correlations among the three facets of orientation to experience and fear of COVID-19, acting with awareness and nonreactivity to inner experience were both negatively related to fear of COVID-19. The items in the Fear of COVID-19 scale were mostly focused on cognition related to exposure to COVID-19. Awareness is a consequence of observation and attention. People practicing awareness may not only experience feelings like fear or anxiety in the present moment (Schmidt, 2011) but may also be prone to rumination (Desrosiers et al., 2013). Hence, people with higher levels of awareness may experience more-intense feelings and thoughts as they encounter catastrophic thinking as a result of exposure to COVID-19. People practicing nonreactivity can openly experience and truly reflect on how they feel in the present moment (Wilson et al., 2003). Nonjudging of inner experience is a component of mindfulness whereby people are encouraged to observe self-critical thoughts and feelings that they experience in the present moment. People may focus less on their self-critical thoughts when treating these thoughts nonjudgmentally.

Our results partially supported our hypothesis that the facets of orientation to experience would be significantly correlated with negative emotions: this was true for nonjudging of inner experience and nonreactivity to inner experience, but not for acting with awareness. Our results showed that the relationship between acting with awareness and a negative emotional state was not statistically significant. However, a previous study found that awareness could be a mediator between mindfulness and depression (Cheung and Ng, 2019). In another study, awareness could predict depression but not anxiety or stress (Cash and Whittingham, 2010). One possible explanation for this discrepancy may be whether or not the participants practice meditation. Our study did not ask the participants whether they practice meditation, so we cannot exclude the effects of meditation on our results. Further studies should examine the effect of meditation on depression.

Our revised model had good goodness-of-fit indices, and both the path from fear of COVID-19 to orientation to experience and from orientation to experience to negative emotion were significant. This result is supported by the study of Bishop et al. (2004). In mindfulness-based practices, orientation to experience is a stage that can help people to shift from negative emotions to normal or peaceful feelings. Despite data showing that orientation to experience can play a crucial role in the regulation of emotions, attention shift and observation are also important stages in preparing to regulate one’s emotions. However, further experimentation and longitudinal studies should be conducted to examine the relationship between mindfulness and emotional regulation.

In conclusion, several limitations were in the present study, one is a cross-sectional study. We could not have a more comprehensive understanding regarding the causal relationship between orientation to experience and mental health. The second limitation is the self-report scale of mindfulness and mental health. We could use the diagnostic interview to examine mental health and mindfulness relationships. The third is the limitation of the instrument. Not all the participants could imagine that they exposure COVID-19 and answer the items on the fear of COVID-19 scale. Thus, people who display fewer imaginations for COVID-19 could not clearly understand the meanings of each item. The last is the lack of a clinical group, the data from the clinical group could truly reflect the experience of mental health and mindfulness.

Despite this study still has some limitations, however, not only confirmed the relationships between fear of COVID-19, orientation to experience, and mental health but also provides preliminary findings concerning the role of nonjudging of inner experience on the regulation of the profile of mood states. Although the nature of this cross-sectional study and the self-reporting scale limit our ability to generalize our results to real-world situations, the study does provide a potential mechanism for demonstrating the effectiveness of mindfulness-based practices in regulating mental health arising as a result of the COVID-19 pandemic. Additionally, we speculate that nonjudging of inner experience may be a crucial component of mindfulness for regulating negative emotions during mindfulness-based meditation.

## Data availability statement

The raw data supporting the conclusions of this article will be made available by the authors, without undue reservation.

## Ethics statement

The studies involving human participants were reviewed and approved by the institute review board of National Tsing Hua University REC no. 11003HT032. The patients/participants provided their written informed consent to participate in this study.

## Author contributions

Y-XL and Y-TC provided the ideas for the work, analyzed and interpreted the data and drafted the work. KL was responsible for writings proposals and grant applications, analyzed data, and wrote the manuscript. All authors contributed to the article and approved the submitted version.

## Funding

This work was supported by YIN SHU-TIEN EDUCATIONAL FOUNDATION (110–8) and College of Education, National Tsing Hua University.

## Conflict of interest

The author declares that the research was conducted in the absence of any commercial or financial relationships that could be construed as a potential conflict of interest.

## Publisher’s note

All claims expressed in this article are solely those of the authors and do not necessarily represent those of their affiliated organizations, or those of the publisher, the editors and the reviewers. Any product that may be evaluated in this article, or claim that may be made by its manufacturer, is not guaranteed or endorsed by the publisher.
